# A novel approach to genetic engineering of T-cell subsets by hematopoietic stem cell infection with a bicistronic lentivirus

**DOI:** 10.1038/s41598-020-70793-6

**Published:** 2020-08-13

**Authors:** N. V. Bogert, J. Furkel, S. Din, I. Braren, V. Eckstein, J. A. Müller, L. Uhlmann, H. A. Katus, M. H. Konstandin

**Affiliations:** 1 Department of Cardiology, University Hospital Heidelberg, Ruprecht-Karls-University, Heidelberg, Germany; 2 DZHK (German Centre for Cardiovascular Research) Partner Site, Heidelberg/Mannheim, Germany; 3 Vector Core Facility, University Hospital Hamburg-Eppendorf, University Hamburg, Hamburg, Germany; 4 DZHK (German Centre for Cardiovascular Research) Partner Site, Hamburg, Germany; 5 Department of Hematology, University Hospital Heidelberg, Ruprecht-Karls-University, Heidelberg, Germany; 6 Institute of Medical Biometry and Informatics, University Hospital Heidelberg, Ruprecht-Karls-University, Heidelberg, Germany

**Keywords:** Genetic engineering, Genetic techniques, Bone marrow transplantation, Haematopoietic stem cells, T cells

## Abstract

Lentiviral modification of hematopoietic stem cells (HSCs) paved the way for in vivo experimentation and therapeutic approaches in patients with genetic disease. A disadvantage of this method is the use of a ubiquitous promoter leads not only to genetic modification of the leukocyte subset of interest e.g. T-cells, but also all other subsequent leukocyte progeny of the parent HSCs. To overcome this limitation we tested a bicistronic lentivirus, enabling subset specific modifications. Designed novel lentiviral constructs harbor a global promoter (mPGK) regulating mCherry for HSCs selection and a T-cell specific promoter upstream of eGFP. Two T-cell specific promoters were assessed: the distal Lck—(dLck) and the CD3δ-promoter. Transduced HSCs were FACS sorted by mCherry expression and transferred into sublethally irradiated C57/BL6 mice. Successful transplantation and T-cell specific expression of eGFP was monitored by peripheral blood assessment. Furthermore, recruitment response of lentiviral engineered leukocytes to the site of inflammation was tested in a peritonitis model without functional impairment. Our constructed lentivirus enables fast generation of subset specific leukocyte transgenesis as shown in T-cells in vivo and opens new opportunities to modify other HSCs derived subsets in the future.

## Introduction

Lentiviral modification of hematopoietic stem cells (HSC) is a valuable tool to investigate inflammatory responses in vivo and has also been used as a tool for therapeutic intervention in patients^1, 2^. Development of inflammatory disease in standardized murine models can take weeks to several months^3, 4^, therefore the bone marrow (BM) in these models must be reconstituted with long-term HSCs (LT-HSCs) to allow for longitudinal follow-ups. To avoid competition of genetic-engineered HSCs with non-modified HSCs, enrichment of lentiviral infected HSCs is necessary prior to reconstitution of the ablated BM. For this purpose pharmacological selection models (e.g. puromycin resistance) or physical sorting using fluorescent protein overexpression have been applied in the past^5–7^. For these selection processes global promoters with ubiquitous activity are employed, causing the target protein to be expressed in the subpopulation of interest (e.g. T-cell), but also in LT-HSCs and all descending daughter cells with the respective global promoter activity. This leads to the criticism that the observed biological effects may be attributed to all leukocyte subpopulations expressing the target protein and not necessarily the subpopulation in question. Conversely, infection of LT-HSCs with a virus regulating the protein of interest by a subset specific promoter will prevent enrichment of successfully transduced multipotent LT-HSCs only. Bicistronic lentiviral vectors have been used in the past successfully in vivo for genetic modifications of cells. Here we introduce a novel lentiviral construct utilizing a bicistronic lentiviral vector harboring two promoters: a global promoter (mPGK) for the expression of the selection marker (mCherry) and a second T-cell specific promoter (either dLck or CD3δ) expressing eGFP to track T-cell subsets. A dual promoter expression cassette with a cell specific second promoter permits definitive lineage specific transgenesis to investigate mechanistic and biological relevance of hematopoietic subsets in inflammatory disease models.

## Material and methods

### Lentiviral construct

**Figure 1:**
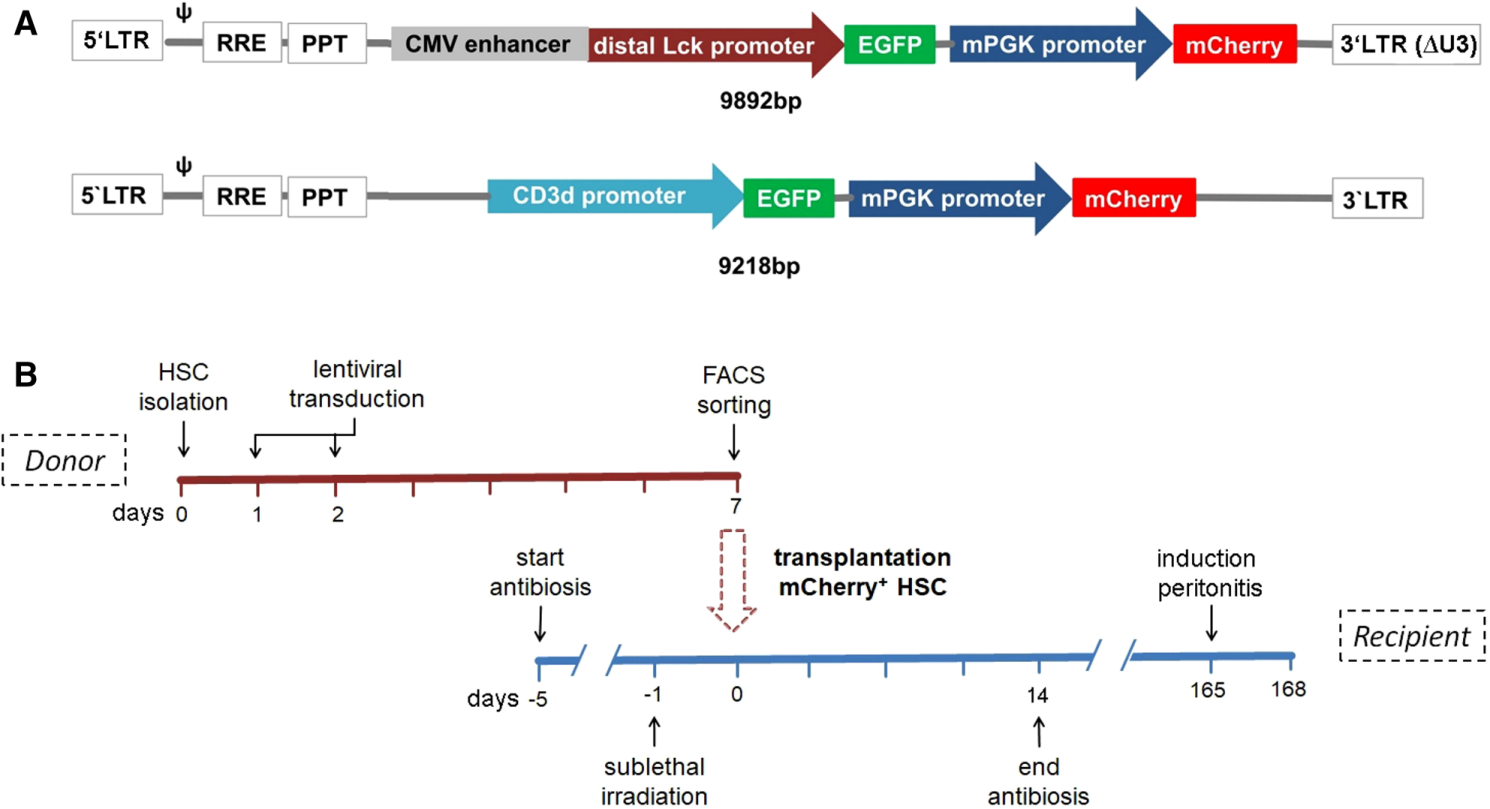
Schematic overview of lentiviral constructs and experimental study design. (**A**) A bicistronic lentivirus: the global mPGK-promoter drives mCherry expression in all transduced cells, whereas the dLck- or CD3δ-promoter regulates eGFP-expression restricted to T-cells (cell-specific-promoter). (**B**) Experimental design: CD105^+^/Sca-1^+^ enriched BM cells were isolated from donor mice, transduced with the lentiviral constructs at day 1 and 2, followed by an enrichment of mCherry^+^ cells by FACS-sorting on day 7. Irradiated recipient mice were transplanted with mCherry^+^ HSCs. 24 weeks after HSC-transplantation. Thioglycolate mediated peritonitis was induced and samples were harvested for flow cytometric analysis 3 days later.

Designed bicistronic lentiviral vectors harbor two constructs driven by two independent promoters. A schematic overview of the two used lentiviral vectors is presented in Fig. 1A. mPGK was used in both constructs as a global promoter located towards the 3′ end and expressing mCherry, which allows identification of successfully transduced cells. Since the global promoter is active in every cell—progenitor, T-cells and non-T-cells—successfully transduced HSCs and all their descendent daughter cells will be mCherry positive, which will allow their enrichment with fluorescence activated cell sorting (FACS) based techniques. The second T-cell specific promoter is located towards the 5′ end and was either the CMV enhanced distal Lck-promoter (dLck; 9892 bp)^8^ or the CD3δ-promoter (9218 bp)^9^ each controlling eGFP expression. Therefore, only committed cells with activity of the T-cell specific promoter will turn green, while non-T-cells and progenitor cells will be eGFP negative.

### Construction of lentiviral vectors and production of lentivirus stocks

As a lentiviral transfer plasmid backbone with eGFP under control of the distal lymphocyte-specific protein tyrosine kinase (dLck) promoter “pLckE-GFP-miR-shRNAscrambled” was ordered from Genecopoiea. To introduce mCherry as a constitutively expressed transduction control, the plasmid was digested with EcoRV and MluI. The murine phosphoglycerate promoter (5′-TGCCATCCAGCTGATATCGGGTAGGGGAGGCG and 5′-CTCACCATCGAAAGGCCCGGAGATGAGGAAGAG) and mCherry (5′-CCTTTCGATGGTGAGCAAGGGCGAG and 5′-attgttccagacgcgtTTACTTGTACAGCTCGTCCATGCC) were amplified by PCR and introduced into the digested vector by InFusion Cloning Kit (Takara Clontech) to generate plasmid pLckE-GFP mPGK-mCherry. For all PCR reactions, PrimeStar GLX polymerase (Takara Clontech) was used. To insert GFP under control of the T-cell surface glycoprotein CD3δ chain (CD3δ) promoter, the resulting plasmid was then digested with EcoRI. Promoter clone #MPRM42268-PF02 was obtained from Genecopoiea and CD3δ-eGFP was used as a template for PCR. The following primer pair was employed: (5′-ggagaagcatgaattTTCGAAGGAGATAGAACCAGATCTTGGAA and 5′-AAAGCTGGGTGAATTCTCGAGCGGCCGCC) and the fragment was introduced into the digested vector by InFusion Cloning Kit (Takara Clontech) to generate plasmid pCD3δ-eGFP mPGK-mCherry. A stock of VSV-G pseudotyped viral particles was produced at the Vector Facility of the University Medical Center Eppendorf (Hamburg, Germany) using lentiviral packaging plasmids psPAX2 (Addgene plasmid #12260) and pMD2.G (Addgene plasmid #12259). After concentration by ultracentrifugation for 2 h at 4 °C (25,000 rpm, SW32Ti rotor) on a 20% sucrose cushion, the pellet was resuspended in DPBS. The functional titer was determined by transduction of HEK293T and quantification by flow cytometry (FACS CantoII, BD Biosciences; FITC Channel): CD3δ lentivirus (1.36 × 10^8^ TU/HEK293) and dLck lentivirus (1.05 × 10^9^ TU/HEK293).

### T1934.4 culture

The immortalized murine hybridoma T-cell line T1943.4 was a kind gift from Dr. Hämmerling, DKFZ University Heidelberg^10^. Cells were cultured in RPMI 1640 (Sigma) + 10% FCS (Gibco) and lentiviral transduction was performed with a multiplicity of infections (MOI) of 50. Flow cytometric analysis was performed 7 days after successful lentiviral transduction. Dead cells were excluded through a viability staining (FVS, BD Bioscience). All used antibodies within the experiments and associated suppliers are listed in the Suppl. Table S1 including final concentrations.

### Isolation and transduction of murine primary T-cells

Murine spleens were harvested from 12 to 14 week adult C57BL/6 mice, crushed and passed through a 40 µm filter as described recently^11^. After red blood cell lysis with Ack-lysis-buffer (0.15 M NH_4_Cl, 10 mM KHCO_3_, 0.1 mM EDTA, ph 7.4), lymphocytes were isolated through density gradient centrifugation with Histopaque 1077 (Sigma). Isolated cells were washed twice with PBS and were resuspended in RPMI 1640 (Sigma) + 10% FCS (Gibco) + 50 µM 2-Mercaptoethanol (Gibco) + 30 IE/ml IL-2 (PeptroTech). Cells were stimulated with a 1:1 ratio of cells and Dynabeads CD3xCD28 (ThermoFisher) according to the manufacturers’ instructions. Lentiviral transduction with an MOI of 50 was performed 24 h after cell stimulation with Dynabeads. Three days later, cells were stained with anti-CD3 antibody and FVS prior to flow cytometric analysis (FACS CantoII, BD Bioscience).

### Isolation and lentiviral transduction of hematopoietic stem and progenitor cells

Bone marrow of 6–8 weeks old C57BL/6 donor mice were washed out of the tibia and femur and were passed through a 40 µm filter as described recently^12^. After red blood cell lysis with Ack-lysis buffer, bone marrow cells were stained with anti-Sca-1-PE and anti-CD105-FITC^13^ (Miltenyi Biotec). Subsequently samples were treated with secondary antibodies coupled to magnetic beads directed against the aforementioned primary antibodies according to the manufacturers’ instructions (Miltenyi Biotec). HSC isolation was performed through double positive selection through magnetic column enrichment using the MACSMultiSort-technique (Miltenyi Biotec). Successful enrichment of hematopoietic stem and progenitor cells was monitored by flow cytometric analysis. Isolated cells were resuspended in StemPro34 Medium (Gibco) supplemented with StemProNutrient (Gibco), 50 ng/ml SCF (R&D Systems), 50 ng/ml TPO (R&D Systems), 12.5 ng/ml Flt3L (R&D Systems) and cultured in round bottom plates at a cell density of 10.000 cells/well. Lentiviral transduction was performed twice during the following two days with an MOI of 50 and cells were kept in culture for the following 6 days. To analyze expression of mCherry in cell subtypes, cells were stained with anti-cKit, anti-Sca1, anti-CD34 and lineage detection cocktail prior to flow cytometric analysis. HSC-subtypes were divided into oligopotent progenitors (OPP): Lin^−^, cKit^+^, Sca1^−^, short-term HSC (ST-HSC): Lin^−^, cKit^+^, Sca1^+^, CD34^+^ and long-term HSC (LT-HSC): Lin^−^, cKit^+^, Sca1^+^, CD34^−^^14, 15^. For HSC-transplantation, cells were isolated through fluorescence activated sorting gated on mCherry^+^ cells using a FACS Aria III from BD Bioscience.

### Bone marrow depletion and HSC transplantation

10–12 weeks old C57BL/6 mice were sublethally irradiated with 7.5 gy with a gamma-irradiator for bone marrow ablation. 24 h after irradiation, sorted mCherry^+^ CD105^+^/Sca-1^+^ enriched BM-cells (1.5–2 × 10^5^ cells/mouse) were applied via tail-vein injection. To prevent infectious complications, drinking water of the mice was supplemented with 50 mg/kg KG/d Ciprofloxacin (Fresenius Kabi) 5 days prior of the irradiation and 14 days post HSC-transplantation.

### In vivo flow cytometric analysis after HSC transplantation

6–8 weeks after HSC-transplantation blood was drawn from mice through submandibular punctuation of V. facialis^16^. Erythrocytes were lysed with one wash step in Ack-lysis-buffer and cells were stained with FVS, anti-CD3, anti-CD44 and anti-CD62L (Suppl. Table S1). T-cell subtypes were classified as CD3^+^/CD44^+^/CD62L^−^ effector T-cells and CD3^+^/CD44^−^/CD62L^+^ naïve T-cells. Analyses were performed on FACS CantoII from BD Bioscience.

### Peritonitis model

A sterile peritonitis was induced with intraperitoneal injection of thioglycolate, 24 weeks after successful HSC-transplantation as described recently^17^. Figure 1B shows a schematic overview of the performed experiment using a timeline. Mice were sacrificed 72 h after induction of peritonitis. The investigation group received mCherry^+^ HSCs transduced with either the lentiviral construct with dLck-promoter (n = 4) or CD3δ-promoter (n = 3). Abdominal cave was flushed with cold PBS supplemented with 2 mM EDTA, 2% FCS to harvest abdominal leukocytes; additionally peripheral blood cells and bone marrow cells were harvested for each animal as described above. Red blood cell contamination was reduced by Ack lysis-buffer incubation. Isolated cells were stained with FVS, anti-CD45, anti-CD3, anti-CD19, anti-Ly6G and anti-CD11b (Suppl. Table S1). Cells were classified as T-cells (CD45^+^/CD3^+^), B-cells (CD45^+^/CD3^−^/CD19^+^), myeloid cells (CD45^+^/CD11b^+^) and granulocytes (CD45^+^/CD11b^+^/Ly6G^+^). Cells of every sample were visually counted using a Neubauer chamber and total peripheral blood cell count was extrapolated to a total volume of 2 ml blood per mouse as published recently^18^. Numbers of leukocyte subtypes were calculated based on flow cytometric analysis (FACS CantoII, BD Bioscience). Recruiting index was calculated as a ratio of the sample cell count compared to blood cell count extrapolated to a total blood volume of 2 ml/mouse (e.g. peritoneum vs. blood or bone marrow vs. blood) as previously described^18^.

### Statistical analysis

Data are indicated as mean ± standard deviation (SD) and were analyzed using Prism 7 software (Graph Pad). Group differences were determined using ANOVA and post-hoc Dunn’s multiple comparison test if more than 2 groups were compared. For statistical analysis of only two groups student t-test or Wilcoxon–Mann–Whitney-test were performed accordingly. Gaussian-distribution was tested prior to analysis by Kolmogorov–Smirnov test. Differences of p < 0.05 were considered statistically significant.

### Ethical approval

All conducted animal experiments of our study were reviewed and approved by the Regierungspräsidium Karlsruhe (G116/15, G26/19). Experiments are respecting all relevant regulatory standards.

## Results

### Confirmation of functionality of the bicistronic lentiviral constructs in vitro

**Figure 2:**
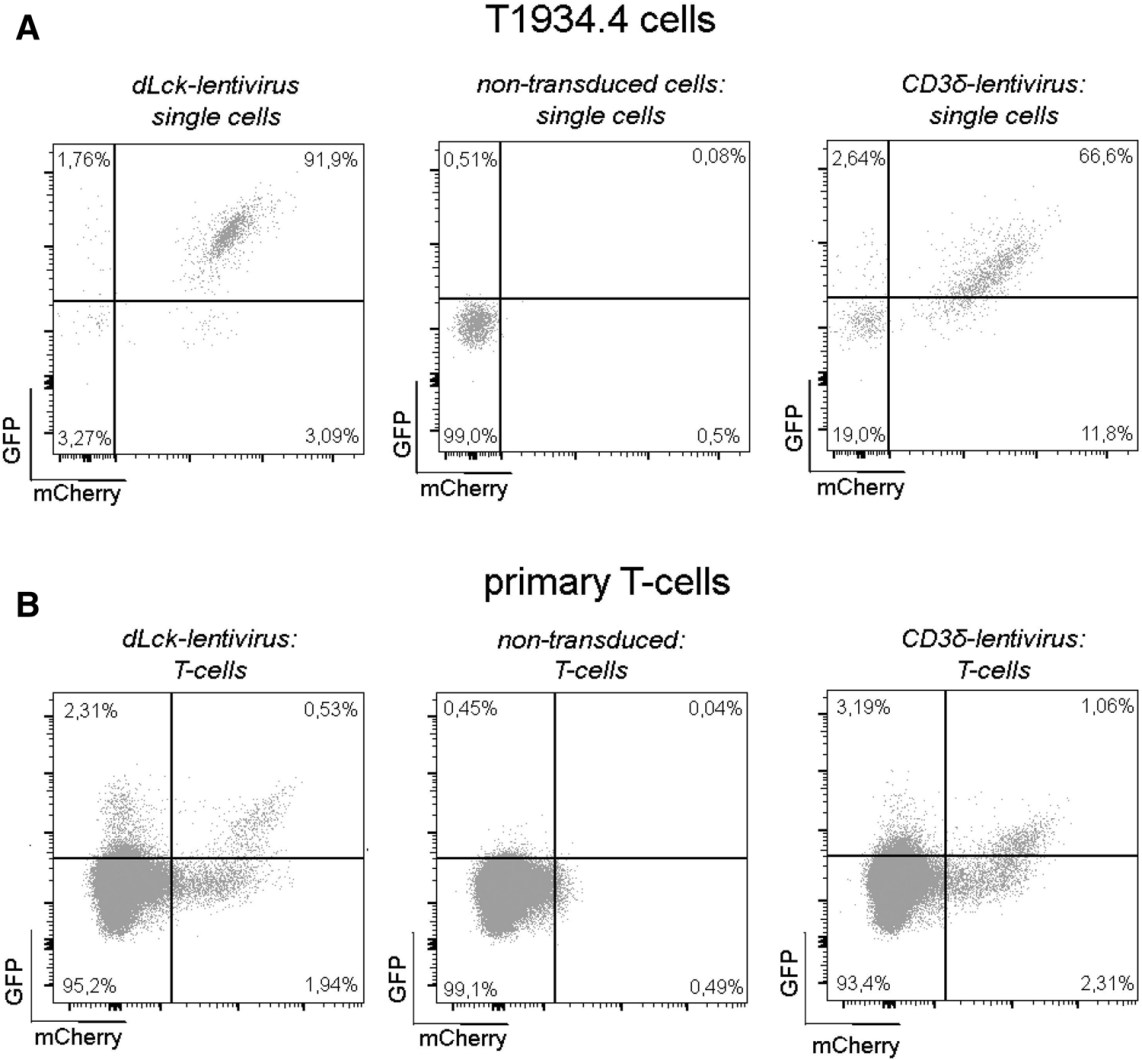
Functionality of bicistronic lentiviral constructs in vitro. (**A**) T1934.4 cells were transduced using MOI 50 with the dLck-lentivirus or the CD3δ-lentivirus. eGFP and mCherry expression were analyzed 7 days post lentiviral transduction. Transduced cells (right and left) are double positive for eGFP and mCherry compared to non-transduced cells (middle). (**B**) Primary murine spleen T-cells were infected with the respective lentivirus as depicted and expanded in vitro using CD3xCD28 costimulation for 3 days in a single transduction experiments. The dot plots depict eGFP and mCherry expression for dLck-lentivirus (middle) and CD3δ-virus (right) treated T-cells gated on mCherry positive and negative subpopulations.

To assess the bicistronic lentiviral approach, the global mPGK-promoter driving mCherry expression, was paired with the dLck-promoter or the CD3δ-promoter regulating eGFP expression, respectively. dLck and CD3δ are T-cell specific markers, enabling identification of T-cells by eGFP expression (Fig. 1A). To test the overall functionality of both viral constructs, we infected the T-cell hybridoma line T1934.4 in vitro. Following 7 days post infection, 92% of the cells expressed mCherry and eGFP when utilizing the dLck-promoter (Fig. 2A, left) whereas, 67% of the cells were double positive for mCherry and eGFP with lentivirus containing the CD3δ-promoter (Fig. 2A, right). To confirm promoter activity in primary murine T-cells, spleen lymphocytes from 12 weeks old mice were isolated by density gradient centrifugation and transduced using the bicistronic lentivirus carrying the dLck or CD3δ-promoter, respectively. Infected T-cells were further expanded for 3 days by CD3 and CD28 costimulation. Flow cytometry analysis of CD3^+^ T-cells showed only 2–3% of cells were positive for mCherry (Fig. 2B, right and left) however, up to 22% of the CD3^+^/mCherry^+^ cells expressed eGFP when employing the dLck-lentivirus (Fig. 2B, left). Additionally, CD3δ-lentivirus infected cells showed 32% eGFP expression in the CD3^+^/mCherry^+^ cell population (Fig. 2B, right). Although transduction efficacy of primary murine T-cells is low, functionality of the lentivirus construct was confirmed. The complete gating strategy is shown in supplementary Fig. 1A,B.

### Efficacy of lentiviral transduction in HSCs in vitro

**Figure 3:**
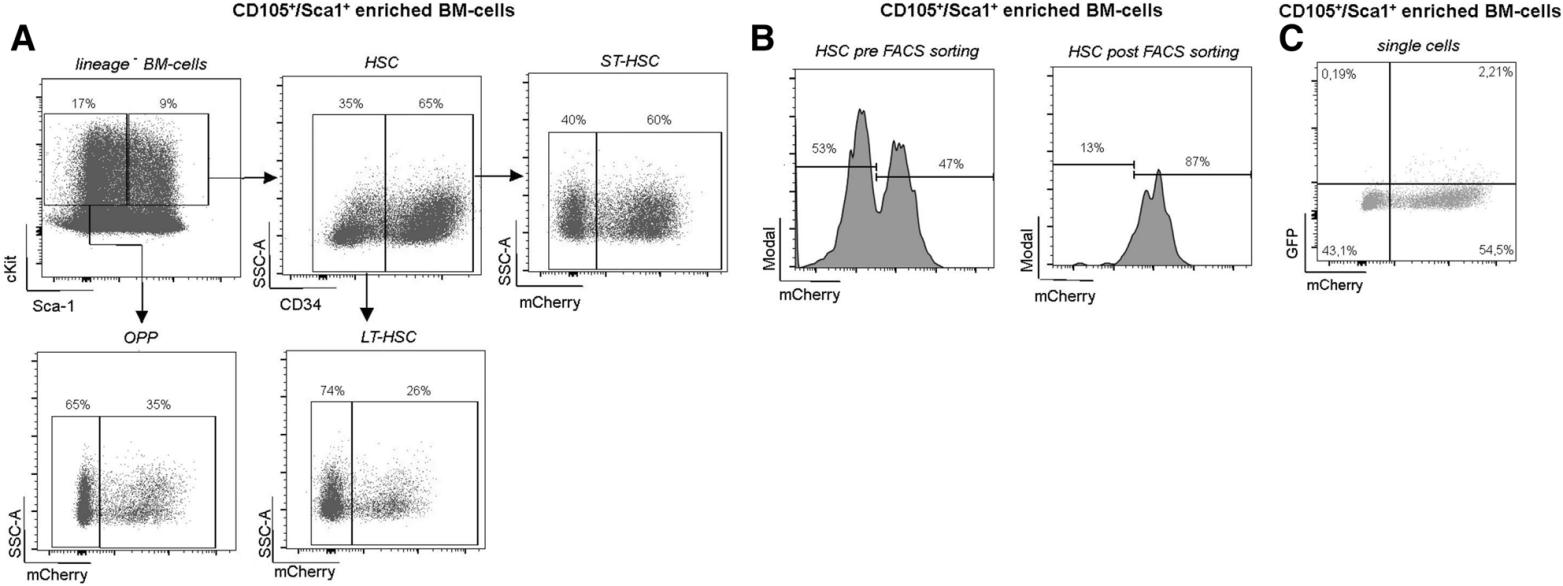
Efficiency of lentiviral transduction and cell sorting. MACS enriched CD105^+^/Sca1^+^ BM-cells were transduced with an MOI of 50 using the dLck-lentivirus. (**A**) Flow cytometric analysis was performed 7 days later to determine expression efficiency. Oligopotent progenitor cells (OPP), short term HSC (ST-HSC), and long term HSCs (LT-HSC) show ubiquitous mCherry expression with a higher mCherry expression rate in ST-HSC. (**B**) mCherry expression is analyzed before (left) and after (right) FACS based cell sorting for transgene CD105^+^/Sca1^+^ BM-cells. (**C**) eGFP was not detectable on transduced mCherry^+^ CD105^+^/Sca1^+^ BM-cells compared to non-transduced cells.

In order to examine the functional significance of hematopoietic subsets in inflammatory disease, multipotent LT-HSCs must be successfully transduced and highly enriched prior to transplantation to avoid competition with non-modified HSCs in vivo. To prevent competition from non-infected HSCs, the experimental approach in Fig. 1B was followed. To address low transduction efficiency from a single round of lentiviral exposure (< 20%, data not shown)^19, 20^, HSCs were transduced twice: on day 2 and on day 3 post isolation with MACS. Six days succeeding lentiviral exposure, engineered cells were enriched via mCherry expression using flow cytometric analysis and transferred via tail vein injection into sublethally irradiated mice. HSC transfer was performed under antibiotic treatment to prevent infection from the compromised immune system. Twenty-four weeks following LT-HSCs transplantation, the experimental peritonitis was initiated by thioglyoclate injection (Fig. 1B). Phenotypically HSCs do not express lineage markers and are double positive for Sca1 and cKit (LSK, lineage^−^, Sca1^+^, cKit^+^). Furthermore, CD34^−^ in these LSK cells identifies LT-HSCs, whereas short-term HSCs are CD34^+^ (ST-HSC). Furthermore, lin^−^, cKit^+^, Sca1^−^ cells are considered oligopotent progenitor cells (OPP)^14, 15^. Expression of mCherry in the aforementioned HSC-subtypes was monitored through flow cytometric analysis seven days post lentiviral transduction in vitro (Suppl. Fig. S2). LT-HSCs displayed a transduction efficiency by mCherry analysis of 26% compared to OPP with 35% positive cells, whereas ST-HSCs were approximately 60% mCherry^+^ (Fig. 3A). To avoid reconstitution of the BM with genetic non-modified HSCs and attenuating the effects of the transgene protein of interest in the respective in vivo model, fluorescence activated sorting of mCherry^+^ cells was performed prior to HSC-transplantation. As shown in Fig. 3B overall mCherry^+^ BM progenitor cells were enriched from about 47% to 87% (Fig. 3B) and show no relevant GFP expression (Fig. 3C).

### Functional assessment of the bicistronic lentiviral constructs in vivo

**Figure 4:**
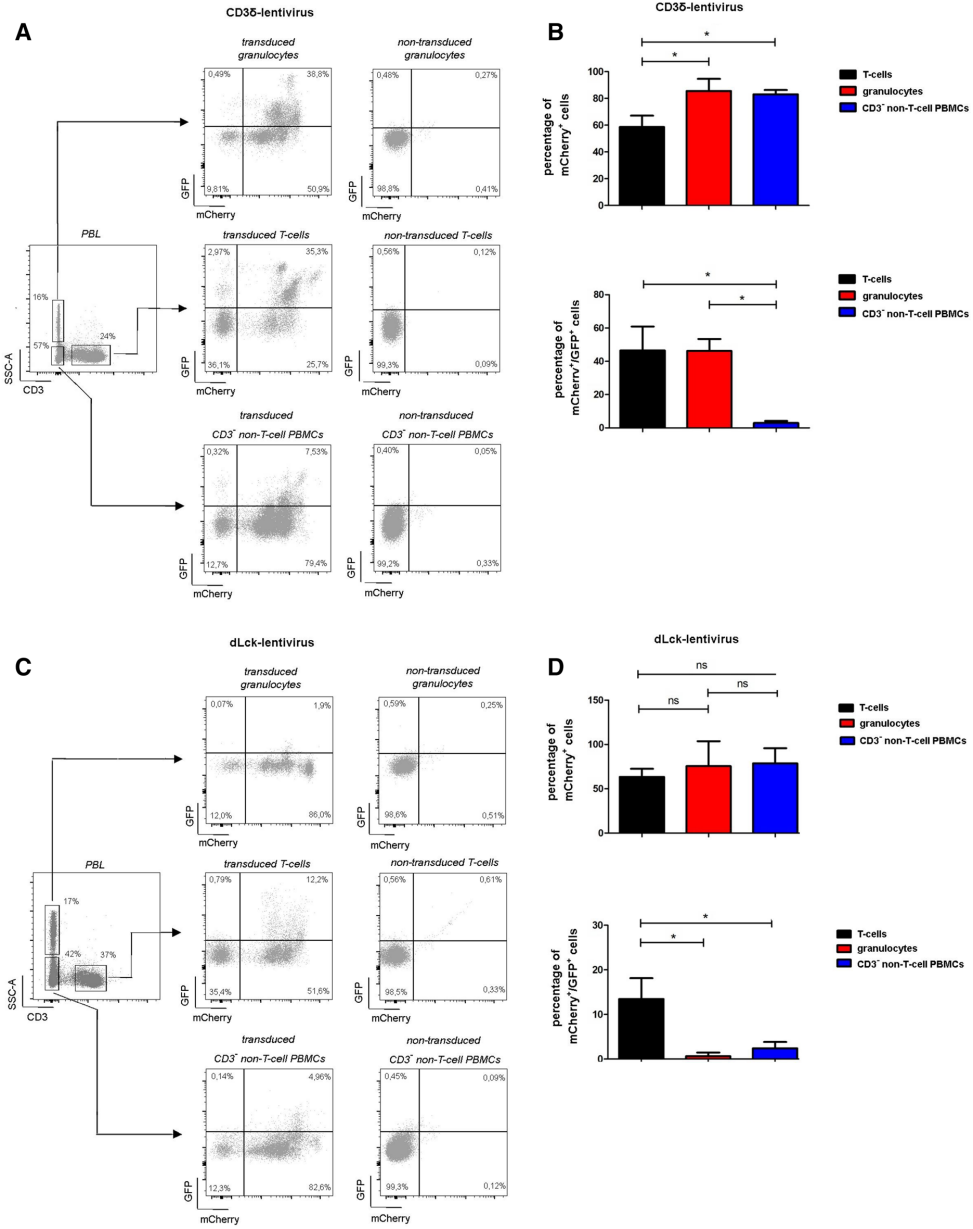
Specificity of lentiviral constructs in peripheral blood. Eight to ten weeks post HSC-transplantation leukocyte subsets in peripheral blood were evaluated by flow cytometric analysis for CD3δ-lentivirus transduced HSCs (n = 5, **A**,**B**) or for dLck-lentivirus transduced HSCs (n = 9, **C**,**D**). Representative dot plots depicting eGFP and mCherry expression are shown for CD3^+^ T-cells **(A**,**C**, left**)**, CD3^−^ non-T-cell PBMCs (**A**,**C**, middle) and CD3^−^ granulocytes (**A**,**C**, right). In (**B**,**D**) quantification of mCherry^+^ and GFP^+^ cells. Error bars indicating SD. *p < 0.05.

**Figure 5:**
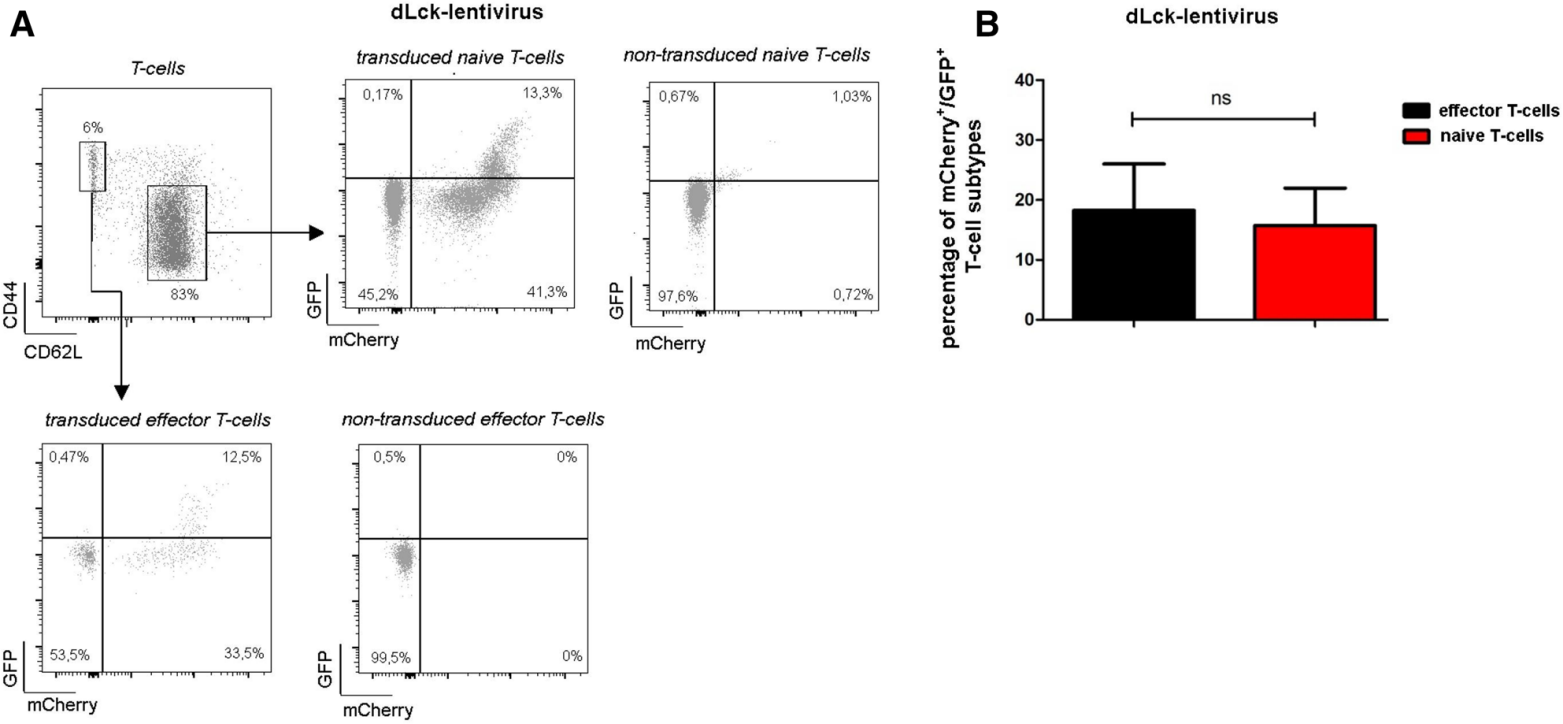
eGFP Expression in T-cell subsets. dLck-promoter driven eGFP and mCherry expression in T-cell subsets was determined by flow cytometric analysis (n = 4). (**A**) Representative dot plots are shown for naïve (left) and memory (right) T-cells. Respective quantification are summarized in (**B**) (naïve vs. memory T-cells). Error bars indicate SD. *p < 0.05.

To assess the performance of our lentiviral constructs in vivo*,* peripheral blood was harvested by facial vein puncture 8–10 weeks post HSC transplantation and stained for CD3 to identify T-cells. Furthermore, according to FSC and SSC properties, viable CD3^−^ leukocytes can be divided into granulocyte population (high SSC properties) and non T-cell peripheral blood mononuclear cell subset (low SSC, CD3^−^ non-T-cell PBMCs). For mice reconstituted with the CD3δ-lentivirus 59 ± 8.5% of all T-cells were mCherry^+^, while 49 ± 14.3% of these cells were also eGFP^+^ (Fig. 4, see also Suppl. Fig. S3A for gating). Whereas, 86 ± 9.0% of the granulocytes and 83 ± 3.2% of the CD3^−^ non-T-cell PBMCs were mCherry^+^. As expected eGFP expression was low in the CD3^−^ non T-cell PBMC population with 3 ± 1.1%, however, of the granulocytes 46 ± 7.2% were also eGFP^+^. In mice with reconstituted BM using HSCs transduced with the dLck-lentivirus 64 ± 9.1% of the T-cells, 76 ± 28.1% of the granulocytes, and 79 ± 17.2% of the CD3^−^ non-T-cell PBMCs were mCherry^+^. 14 ± 4.6% of the T-cells were also eGFP^+^, while granulocytes (0.6 ± 0.8%), and the CD3^−^ non-T-cell PBMCs (2.4 ± 1.4%) minimally expressed eGFP^+^ (Fig. 4C,D, Suppl. Fig. S3B). Since only a fraction of mCherry expressing T-cells were also eGFP positive, we asked whether the dLck-promoter might only be active in a specific T-cell subpopulation. Therefore, experiments were repeated and samples counterstained for naïve CD62L^+^ and memory CD44^+^ T-cells (Fig. 5A,B, Suppl. Fig. S3C). However, none of these subsets showed a preferential eGFP expression.

### Recruitment of lentiviral transduced leukocytes in the sterile peritonitis model

**Figure 6:**
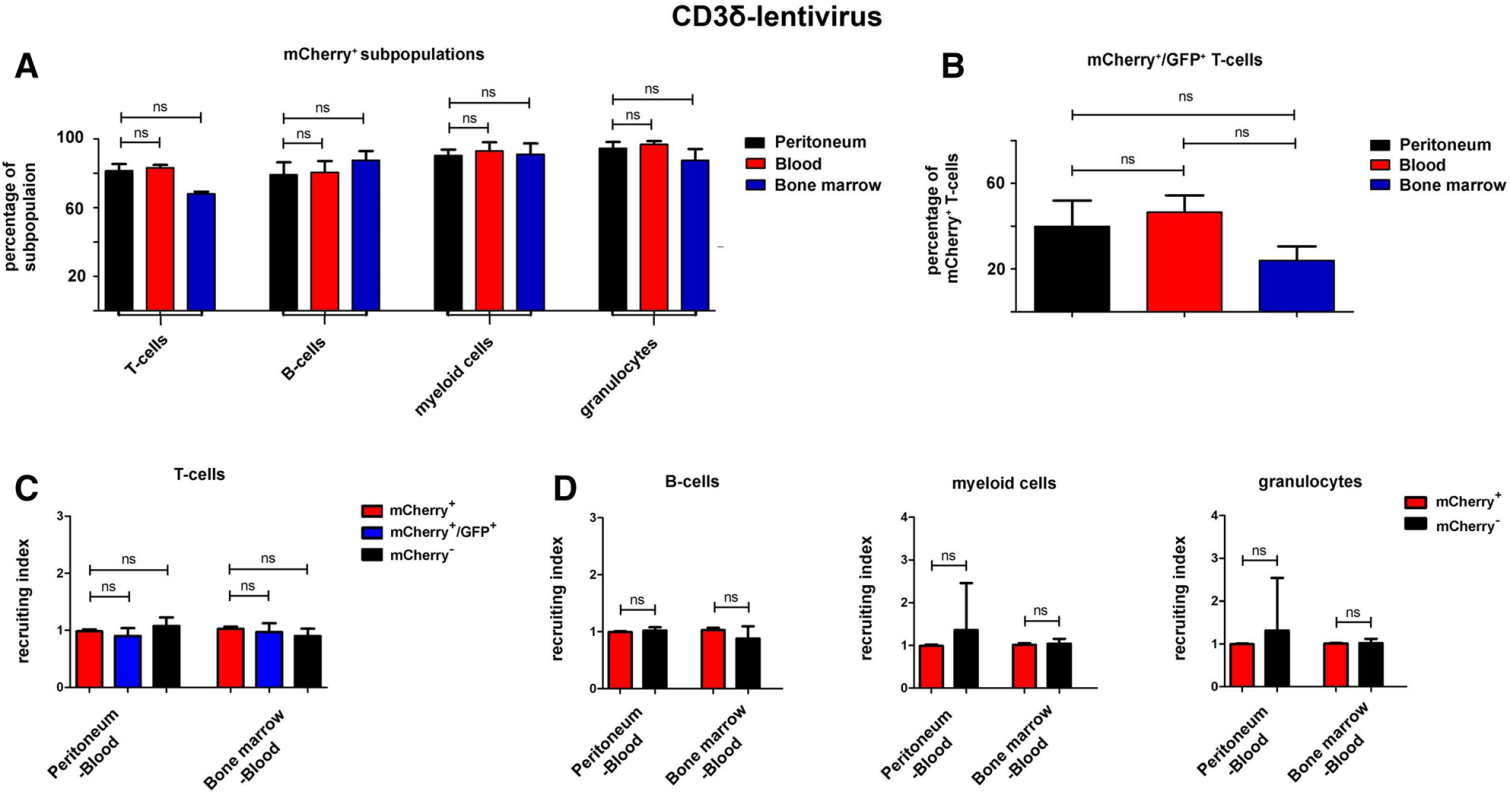
Peritonitis model in mice transplanted with CD3δ-virus transduced HSCs. Sterile peritonitis was induced 24 weeks after transplantation of HSCs transduced with the CD3δ-virus construct (n = 3). (**A**) The percentage of mCherry^+^ cells within the respective leukocyte subset is quantified in peritoneum, blood, and BM. (**B**) The proportion of eGFP expressing T-cells within the mCherry^+^ T-cell population is shown for the same three compartments without significant difference. (**C**) The recruiting index for mCherry^+^ T-cells, mCherry^−^ T-cells, and mCherry^+^/eGFP^+^ double positive T-cells have been calculated between peritoneum-blood, and BM-blood. (**D**) For B-cells (left), myeloid cells (middle) and granulocytes (right) mCherry^+^ and mCherry^−^ cells show no significant difference in the recruiting index peritoneum-blood or BM-blood. Error bars indicate SD. *p < 0.05.

In many murine disease models recruitment of leukocyte subpopulations of interest to the site of inflammation is a critical readout^21–23^. Therefore, we induced a sterile peritonitis 24 weeks following HSC transfer. In Fig. 6A the percentage of mCherry expressing CD3^+^ T-cells, CD19^+^ B-cells, CD11b^+^ myeloid cells and Ly6G^+^ granulocytes is depicted for mice reconstituted with CD3δ-engineered HSCs (the complete gating strategy is shown in Suppl. Fig. S4). Within each leukocyte subpopulation no significant difference could be found when cells were harvested from peritoneum, peripheral blood, or the bone marrow, suggesting no relevant impact of the lentiviral treatment on immune cell trafficking within these compartments (Fig. 6A). Furthermore, the fraction of eGFP^+^ cells within the mCherry^+^ T-cells was similar between all three analyzed compartments (Fig. 6B). To correct for the variability regarding the extent of chimerism of mCherry^+^ and mCherry^−^ cells between different animals the recruiting index (RI) was calculated per animal^18^. As depicted in Fig. 6C, the RIs from blood to peritoneum and from blood to BM was nearly identical, with only minor variability for the subsets of mCherry^+^ T-cells, mCherry^+^/GFP^+^ T-cells, and mCherry^−^ T-cells. Additionally, RI calculations from blood-peritoneum and blood-BM for B-cells, myeloid cells and granulocytes, exhibited no significant differences irrespective of mCherry positivity (Fig. 6D). In vivo experiments were further repeated using the dLck-driven construct (Suppl. Figs. S5, S6). Analogous to the CD3δ modified HSCs , no significant differences between the distribution of mCherry^+^ cells in the peritoneum, blood, and BM was seen for T-cells, B-cells, myeloid cells or granulocytes (Suppl. Fig. S5A). Lastly, in the calculation of the RIs blood-peritoneum and blood-BM, no differences were detectable in any leukocyte subset population analyzed (Suppl. Fig. S5C,D). Taken together, these data suggests that lentiviral modification of LT-HSCs does not impact recruitment properties of leukocyte subpopulations.

## Discussion

Lentiviral modifications of HSCs is a well-established technique, enabling stable and long-lasting genetic modification of all future daughter cells through genomic integration^24, 25^. Conventional mono- or biscitronic lentiviral vectors harbor a global promoter which leads to target expression in every descending daughter cell from transduced HSCs, preventing T-cell subset-specific genetic modification. Our novel lentiviral construct includes employing a bicistronic lentiviral vector, harboring a global promoter (mPGK) for selection of successfully transduced HSCs and a second, cell-specific promoter (dLck or CD3δ) expressing eGFP which enables a cell specific expression e.g. in T-cells. This model opens the field for new applications and allows for the first time a T-cell specific modification with only minor affection of other leukocyte subsets.

dLck and CD3δ are well known promoters with predominant activity in T-cells. Whereas the dLck promoter develops activity after thymocyte T-cell selection^26, 27^, the CD3δ promoter is active in immature CD3^+^ thymocytes prior to thymocyte T-cell selection^28^. Importantly, both promoters show activity in peripheral blood T-cells^9, 26, 27^. Lentiviral constructs incorporating dLck or CD3δ respectively, were tested for functionality in the murine T-cell hybridoma line in vitro. In this cell line, nearly all cells were successfully transduced and displayed simultaneous expression of mCherry along with eGFP, confirming proper expression. In contrast, transduction efficacy of primary spleen T-cells was significantly lower (< 5%) and only about a third of mCherry^+^ T-cells were also eGFP positive. It is well known that murine T-cells are difficult to infect and require complex cell culture conditions, including CD3xCD28 costimulation for proper lentiviral transduction^29, 30^. Furthermore, whether low efficacy of the second T-cell specific promoter (dLck or CD3δ) within the mCherry^+^ compartment is a T-cell specific phenomenon or whether similar low expression will be seen using an alternative leukocyte specific promoter will be explored in future studies and lies beyond the scope of this manuscript.

For in vivo experimentation CD105^+^/Sca-1^+^ enriched BM cells were transduced with lentiviral constructs and FACS sorted prior to HSC transplantation to enrich for mCherry^+^ BM progenitor cells. Eight to ten weeks following transplantation, peripheral blood cells of transplanted mice were analyzed for mCherry and eGFP expression. The dLck-lentivirus showed less than a sixth of mCherry^+^ T-cells were also eGFP positive, whereas utilizing the CD3δ-lentivirus, about half of the mCherry^+^ T-cells were also eGFP positive. This data is aligns with the study published by Zhang et al. using a conventional generated mouse line, exhibiting similar insertion-site dependent weak expression of a dLck-promoter driven eGFP expression in vivo^8^. Nevertheless, the dLck-promoter is highly specific for T-cells and seems to be equally active in T-cell subtypes, such as naïve or effector T-cells. In contrast to the dLck-promoter, the CD3δ-promoter led to a much higher eGFP expression in T-cells, however, surprisingly eGFP expression was also detectable in about 50% of mCherry^+^ granulocytes as well. In contrast to our observations, conventionally generated transgenic mice with CD3δ-promoter driven expression show a high T-cell specificity without any documented activity of the promoter in peritoneal derived Gr1^+^ granulocytes (without induction of a peritonitis)^9^. However, peripheral blood granulocytes in CD3δ driven transgenic mice were not assessed for promoter driven expression^9^. Furthermore, CD3 surface expression has been documented on 10% of murine and human granulocytes^31^. Therefore, our approach may be useful in delineating the biological meaning of this phenomenon in the future and does not represent leakiness or unspecificity of the CD3δ-promoter.

Collectively, the dLck-promoter shows high T-cell specificity however, activity is limited to only a sixth of all T-cells. In contrast the CD3δ-promoter shows stronger expression in the majority of T-cells but, labels CD3 expressing granulocytes. Therefore, depending on the biological question the right promoter may be chosen: e.g. for recruitment studies high specificity in only T-cells is desirable without affecting the ongoing inflammation, therefore, the dLck-promoter should be chosen. In contrast, if the overall effect of manipulated T-cell response upon an inflammatory disease model is under examination strong transgene expression in the majority of T-cells is needed and the CD3δ-promoter should be applied—at the cost that the observed effects might be T-cell and potentially granulocyte mediated.

Extravasation and recruitment of leukocytes into a region of inflammation is a complex multistep process. Functional in vivo assessment of recruitment using our bicistronic lentivirus was done in a sterile peritonitis model through intraperitoneal injection of thioglycolate 24 weeks after HSC transplantation, a standardized procedure leading to induced infiltration of leukocytes into the peritoneal cavity^17^. Lentiviral transduced cells demonstrated no impairment in recruitment compared to non-transduced cells. Although the study’s main focus delineated the effects of leukocyte recruitment following lentiviral modification, the immune system is complex and other functions were not analyzed (e.g. cytokine release, antigen specific response, proliferation, maturation, etc.). However, lentiviral modification of HSCs with global promoters have been applied successfully in the past without causing impairment in immune responses^2^. The recruitment experiments showed similar eGFP expression within the mCherry positive cells in peripheral blood and also in the peritoneum. In the bone marrow compartment, a strong trend of reduced eGFP expression in mCherry positive T cells was seen (Fig. 6B, Suppl. Fig. S5B). Therefore, analysis of this compartment in future studies has to be well controlled with appropriate constructs without the respective gene of interest. A potential explanation may be that the earlier stages of T cell development have a weaker activity of dLck and CD3δ promoters in these less mature cells of the bone marrow, however, this is speculation and requires further investigation.

In this study we establish a novel method for the generation of transgenic leukocyte subpopulations in vivo by lentiviral transduction of HSCs with a bicistronic lentivirus. This model allows for fast generation of transgenic leukocytes in vivo without the need for conventional, time-consuming breeding of transgenic mouse lines, effectively reducing the time and number of experimental animals needed for animal experimentation. The designed lentiviral constructs enable target protein expression in T-cells while maintaining effective recruitment responses in vivo. The ability to swap and replace target promoters or proteins of interest using our bicistronic lentivirus, allows for a personalized construct design to specifically address the investigators biological question. Therefore, a dual promoter lentiviral tool has broad application potential to assess multiple immune compartments and different target proteins in various inflammatory or disease models. Leukocyte subset targeted expression can serve as a potential long-lasting therapeutic approach for genetic disorders in patients like primary immunodeficiencies, severe combined immunodeficiencies (SCIDs), Wiskott-Aldrich syndrome (WAS) or DiGeorge syndrome. Aside from genetic disorders, a lentiviral therapy with cell specific targeted expression in leukocyte subsets can be beneficial in cancer patients and or combined with chimeric antigen receptor (CARs) T-cell therapies.

## Supplementary information

Supplementary Legends

Supplementary Figure S1

Supplementary Figure S2

Supplementary Figure S3

Supplementary Figure S4

Supplementary Figure S5

Supplementary Figure S6

Supplementary Table S1.
