# Supplementary material for: A novel approach to genetic engineering of T-cell subsets by hematopoietic stem cell infection with a bicistronic lentivirus
Source: Sci Rep. 2020 Aug 13;10:13740. doi: 10.1038/s41598-020-70793-6 (PMC7426960; doi:10.1038/s41598-020-70793-6)
Supplement: Supplementary file 1 — Supplementary Legends [file 41598_2020_70793_MOESM1_ESM.docx]

**Supplementary figure legends**

**S-Figure 1:**

**(A)** Complete flow cytometry gating strategy of dLck-lentivirus transduced T1934.4 T-cells for fig. 2A. In **(B)** the respective gating is shown for primary spleen T-cells (see fig. 2B).

**S-Figure 2:**

Complete flow cytometry gating strategy of MACS sorted CD105^+^/Sca-1^+^ enriched BM-cells 6d after lentiviral transduction with dLck-lentivirus (see fig. 3).

**S-Figure 3:**

Complete flow cytometric analysis gating strategy of peripheral blood cells of lentiviral transduced HSCs are shown. **(A)** CD3δ-lentivirus samples were stained for CD3 and gated for T-cells, granulocytes, and CD3^-^ non-T-cell PBMCs (see fig. 4). **(B)** dLck samples were stained for CD3 to evaluate T-cells, granulocytes, and CD3^-^ non-T-cell PBMCs (see fig. 5). **(C)** dLck samples were stained for CD3, CD62L, and CD44 to analyze T-cells, memory T-cells and naïve T-cells (see fig. 5).

**S-Figure 4:**

Flow cytometry gating strategy of harvested cells 75h after induction of peritonitis in mice transplanted with CD3δ-lentivirus transduced HSCs and stained with CD45, CD3, CD19 and CD11b (see fig. 6).

**S-Figure 5:**

Sterile peritonitis was induced 24 weeks following transplantation of HSCs transduced with the dLck-virus construct (n=4). **(A)** The percentage of mCherry^+^ cells within the respective leukocyte subset is quantified in peritoneum, blood, and BM. **(B)** The proportion of eGFP expressing T-cells within the mCherry^+^ T-cell population is shown for the same three compartments. **(C)** The recruiting index for mCherry^+^ T-cells, mCherry^-^ T-cells, and mCherry^+^/eGFP^+^ double positive T-cells has been calculated between peritoneum-blood and BM-blood. **(D)** For B-cells (left), myeloid cells (middle) and granulocytes (right) mCherry^+^ and mCherry^-^ cells show no significant difference in the recruiting index peritoneum-blood or BM-blood. Error bars indicate SD. * p < 0.05

**S-Figure 6:**

Flow cytometry gating strategy of harvested cells 75h after induction of peritonitis in mice transplanted with dLck-lentivirus transduced HSCs and stained with CD45, CD3, CD19 and CD11b (see suppl. fig. 5).
