# Supplementary figures and images for: A novel approach to genetic engineering of T-cell subsets by hematopoietic stem cell infection with a bicistronic lentivirus
Source: Sci Rep. 2020 Aug 13;10:13740. doi: 10.1038/s41598-020-70793-6 (PMC7426960; doi:10.1038/s41598-020-70793-6)

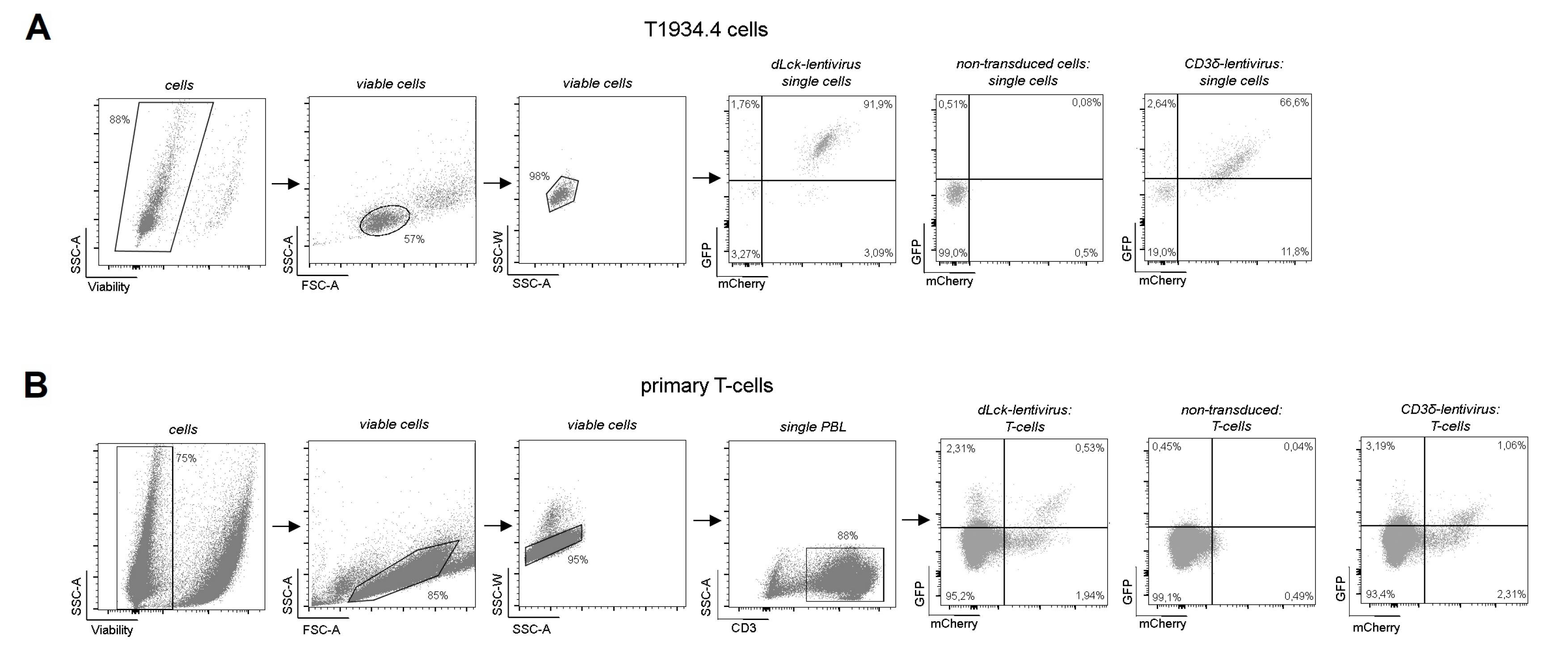

Supplement: Supplementary file 2 — Supplementary Figure S1 [file 41598_2020_70793_MOESM2_ESM.jpg]

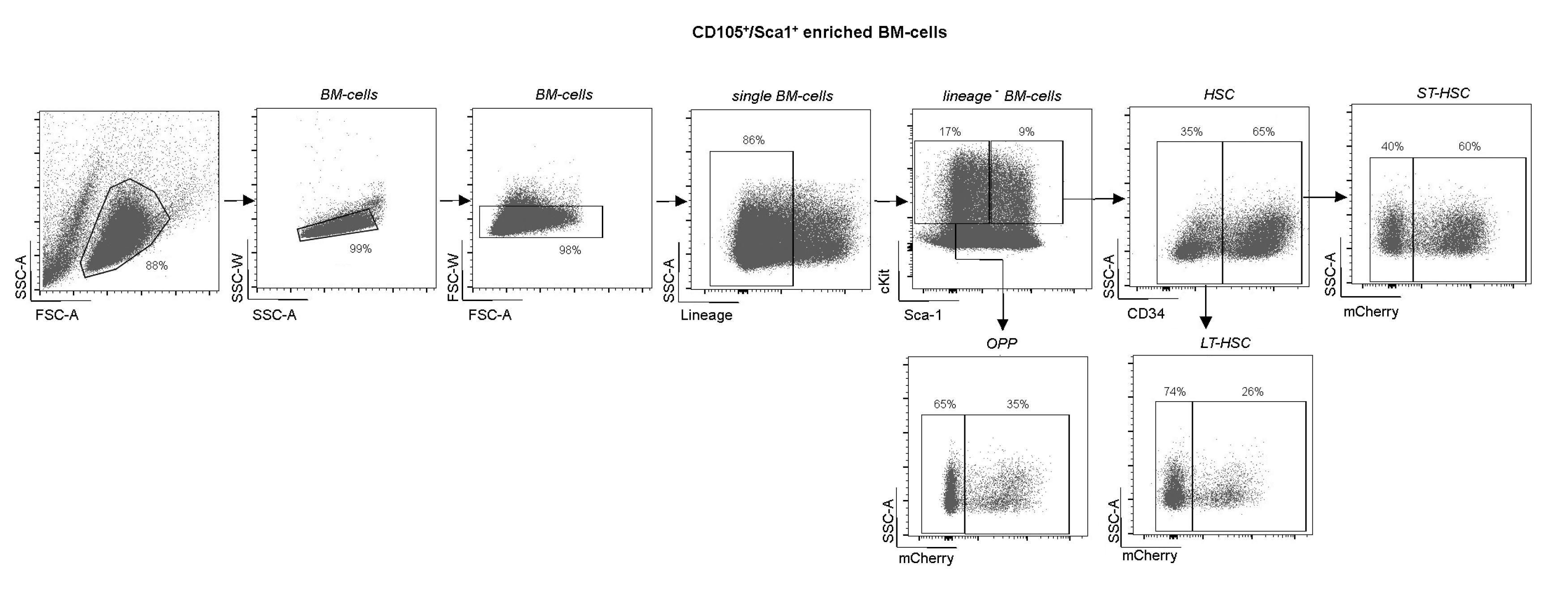

Supplement: Supplementary file 3 — Supplementary Figure S2 [file 41598_2020_70793_MOESM3_ESM.jpg]

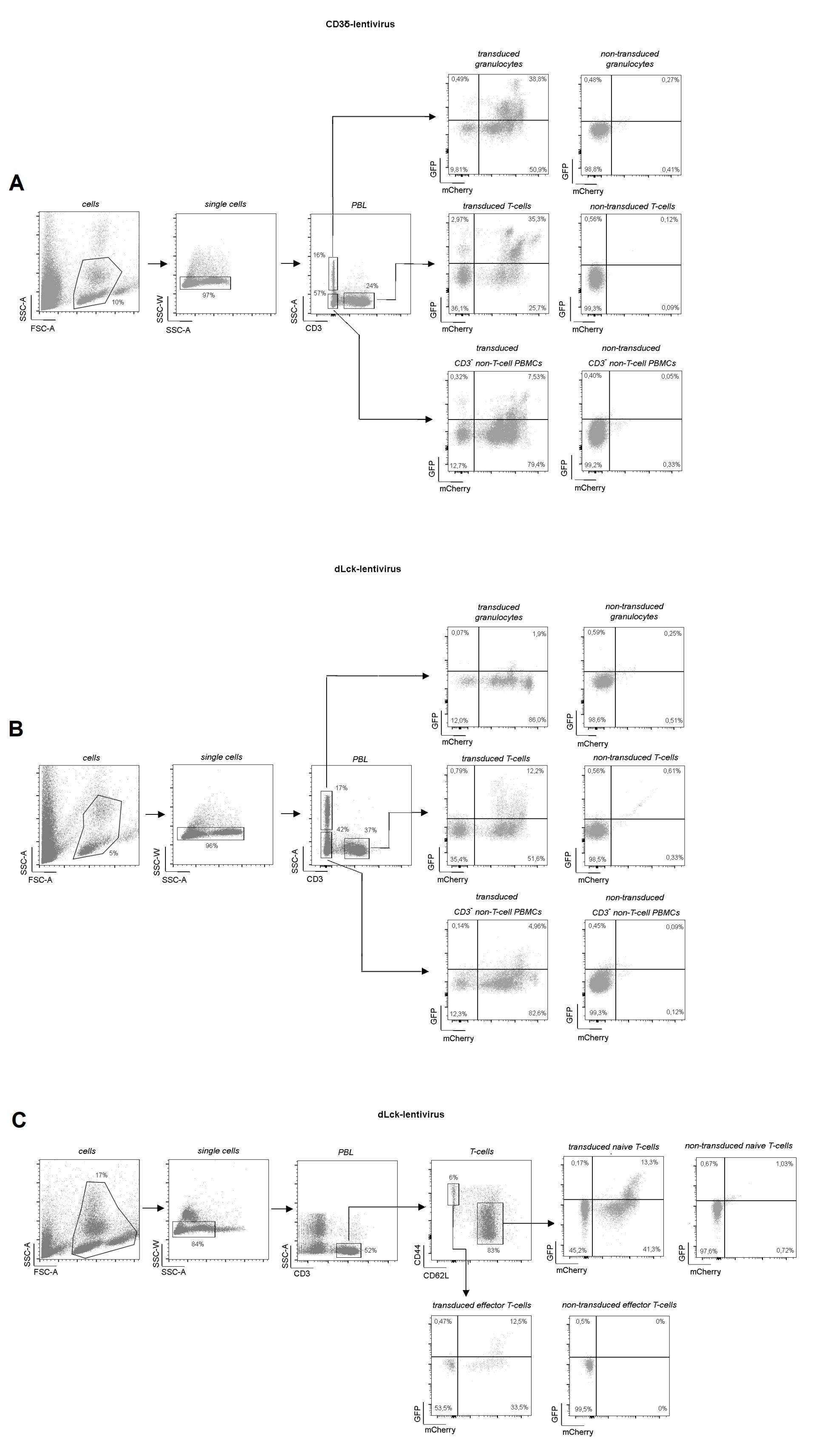

Supplement: Supplementary file 4 — Supplementary Figure S3 [file 41598_2020_70793_MOESM4_ESM.jpg]

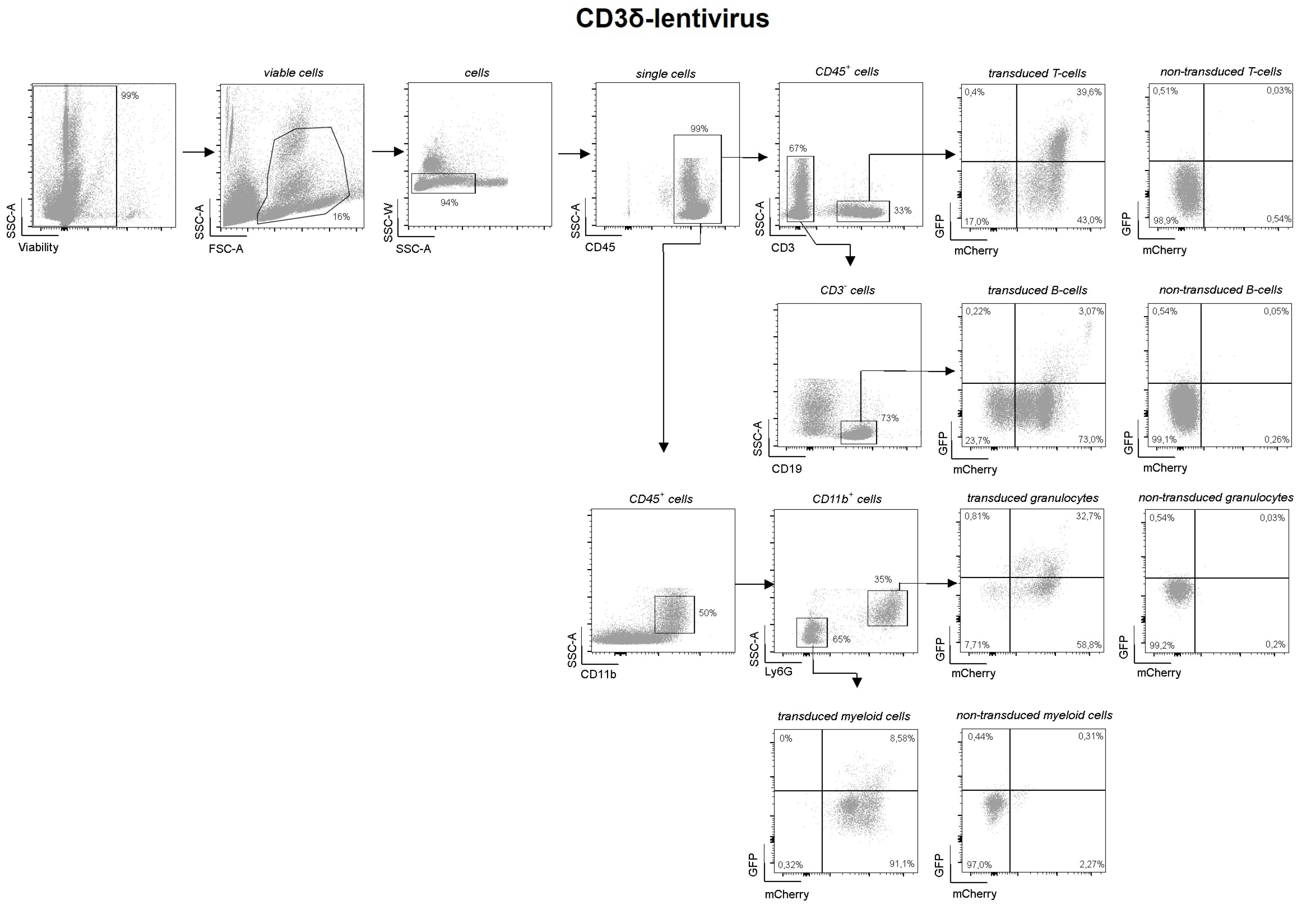

Supplement: Supplementary file 5 — Supplementary Figure S4 [file 41598_2020_70793_MOESM5_ESM.jpg]

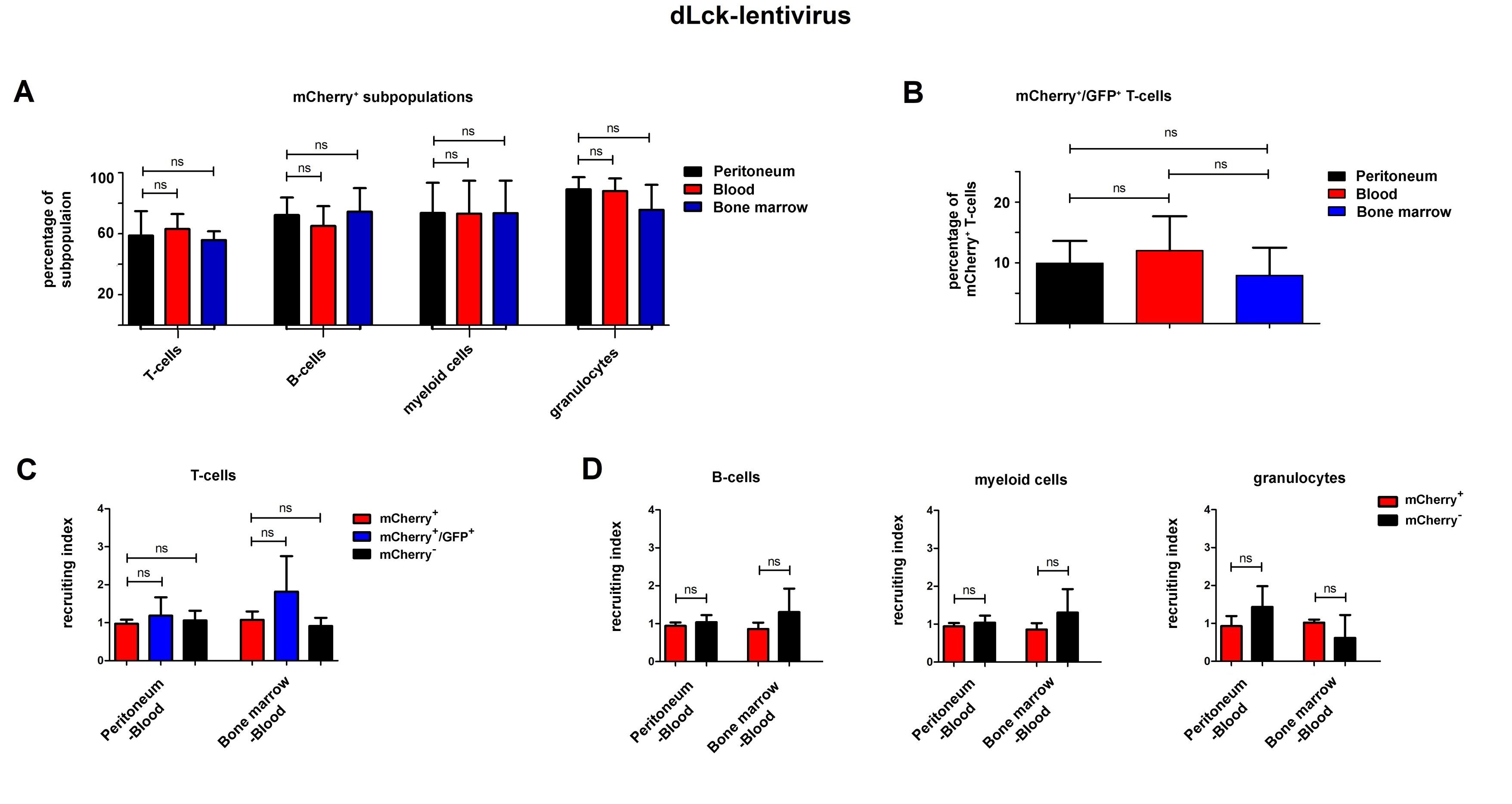

Supplement: Supplementary file 6 — Supplementary Figure S5 [file 41598_2020_70793_MOESM6_ESM.jpg]

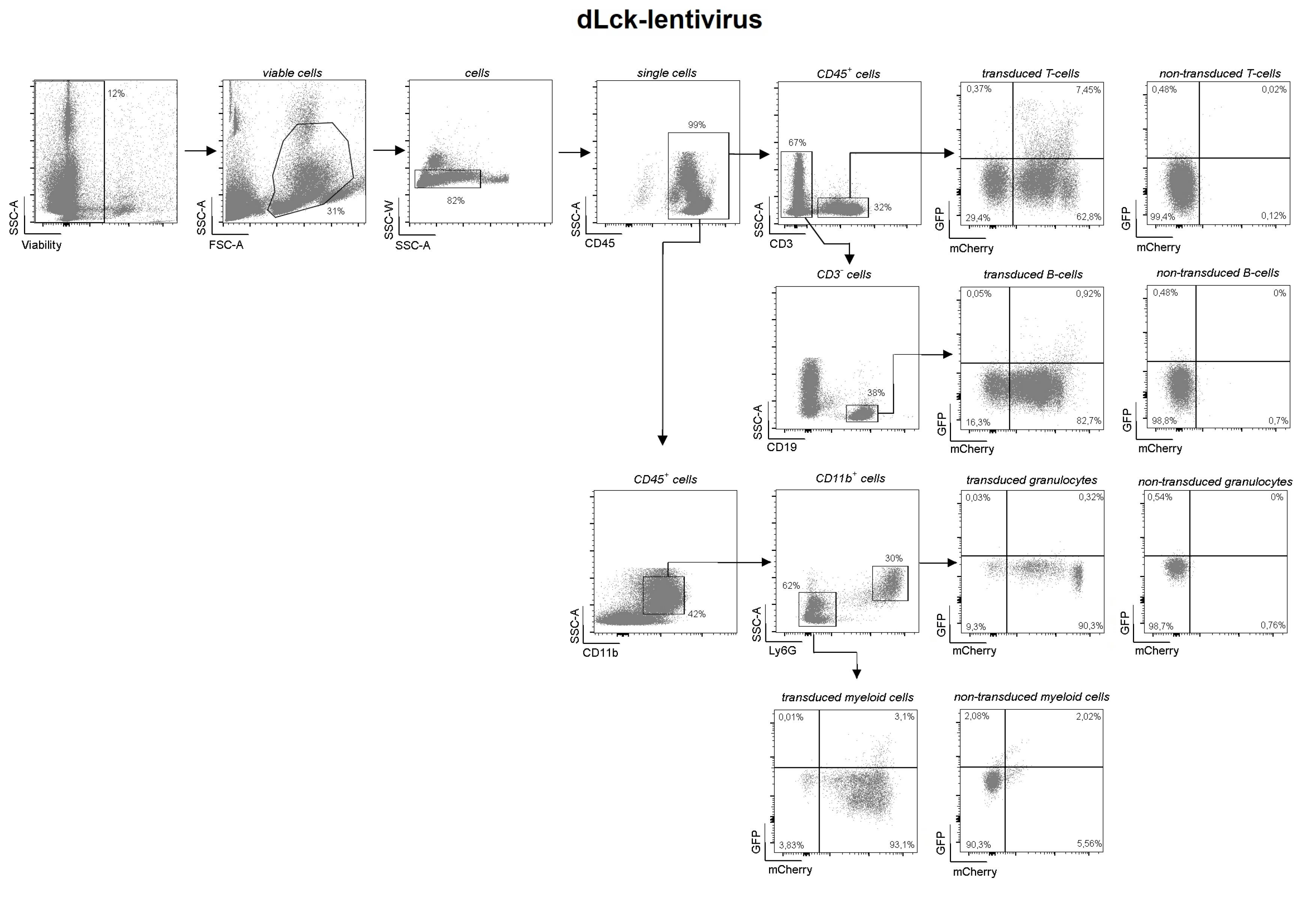

Supplement: Supplementary file 7 — Supplementary Figure S6 [file 41598_2020_70793_MOESM7_ESM.jpg]
