# Supplementary material for: A novel approach to genetic engineering of T-cell subsets by hematopoietic stem cell infection with a bicistronic lentivirus
Source: Sci Rep. 2020 Aug 13;10:13740. doi: 10.1038/s41598-020-70793-6 (PMC7426960; doi:10.1038/s41598-020-70793-6)
Supplement: Supplementary file 8 — Supplementary Table S1. [file 41598_2020_70793_MOESM8_ESM.docx]

**Supplement table 1:**

**Antibodies and kits**

| **name** | **clone** | **article-nr.** | **supplier** | **Dilution** |
| --- | --- | --- | --- | --- |
| anti-CD3 | 17A2 | 564008 | BD Bioscience | 1:100 |
| anti-CD45 | Clone  30-F11 | 561869 | BD Bioscience | 1:100 |
| anti-CD19 | 1D3 | 561738 | BD Bioscience | 1:100 |
| FVS | - | 564405 | BD Bioscience | 1:1500 |
| anti-CD44 | IM7 | 561862 | BD Bioscience | 1:100 |
| anti-CD62L | MEL-14 | 560516 | BD Bioscience | 1:100 |
| Fc-block CD16/CD32 | 2.4G2 | 553142 | BD Bioscience | 1:100 |
| anti-CD11b | M1/70 | 557657 | BD Bioscience | 1:100 |
| anti-Ly6G | 1A8 | 560601 | BD Bioscience | 1:100 |
| Lineage detection cocktail | - | 130-090-856 | Miltenyi Biotec | 1:100 |
| anti-cKit | 2B8 | 553355 | BD Bioscience | 1:100 |
| anti-Sca1 | D7 | 560653 | BD Bioscience | 1:100 |
| anti-CD34 | RAM34 | 553733 | BD Bioscience | 1:100 |
| anti-CD105 MultiSort Kit PE | - | 130-092-924 | Miltenyi Biotec | - |
| anti-Sca1 MicroBeads Kit FITC | - | 130-092-529 | Miltenyi Biotec | - |
